# Cell-Based Therapies for the Treatment of Shoulder and Elbow Tendinopathies: A Scoping Review

**DOI:** 10.1155/2021/5558040

**Published:** 2021-04-24

**Authors:** Berardo Di Matteo, Riccardo Ranieri, Angelo Manca, Simone Cappato, Maurilio Marcacci, Elizaveta Kon, Alessandro Castagna

**Affiliations:** ^1^Department of Biomedical Sciences, Humanitas University, Pieve Emanuele, Italy; ^2^First Moscow State Medical University, Sechenov University, Moscow, Russia; ^3^IRCCS Humanitas Research Hospital, Rozzano, Italy; ^4^Humanitas San Pio X Institute, Milano, Italy

## Abstract

**Introduction:**

Tendinopathies are a common cause of disability among the general population, and their management is challenging due to the degenerative nature of these disorders. The aim of this paper is to perform a scoping review of the available clinical evidence on the application of cell-based therapies for the management of elbow and rotator cuff tendinopathies, in order to summarize the current application methods and to shed light on the therapeutic potential and current limitations of these biologic approaches.

**Materials and Methods:**

A scoping review of the literature was performed on the PubMed and Scopus databases using the following inclusion criteria: clinical reports of any level of evidence, written in English, with no time limitation, on the use of cell-based approaches to treat rotator cuff or elbow tendinopathies, including studies on biological augmentation during the surgical procedure. Exclusion criteria were as follows: case reports or mini case series (<5 patients), articles not written in English, and reviews. Relevant data were then extracted and collected in a single database with the consensus of the two observers to be analyzed for the purposes of the present manuscript.

**Results:**

Seven papers dealing with rotator cuff tears were included. Four of them investigated the effect of injections, either MSCs alone or in combination with PRP, whereas three studies investigated the use of MSCs in combination with surgery. In all cases, an improvement was found in terms of clinical scores, with even evidence of tendon healing documented at second-look arthroscopy. Six papers dealt with elbow tendinopathies: three studies described the use of MSCs either with or without surgery, reporting significant clinical improvement and three studies analyzed the use of different types of cells (collagen-producing cells and autologous tenocytes) and, even in this case, clinical improvement was reported.

**Conclusion:**

All the papers included suggested a beneficial role of cell-based approaches to treat common upper limb tendinopathies, with an overall satisfactory safety profile. However, the lack of high-level evidence and the presence of controversial issues, such as interproduct variability, harvest source, and application strategies, do not allow standardization of these novel biologic approaches, whose efficacy needs to be confirmed with properly designed randomized trials.

## 1. Introduction

Tendon-related problems are one of the main causes of disability in modern times, and even if their prevalence is underestimated, they cause marked reduction in working ability and sport practice.

Tendinopathy is characterized by prolonged pain and is often activity related. Many studies [1–4] have underlined that pathological disorders involving tendons are mainly based on degenerative processes. This is reflected by the presence of nonacute inflammatory cells and the presence of areas of collagen degeneration, myxoid degeneration, and increased ground substance [2, 4]. Aetiology is considered mainly microtraumatic, as a consequence of both repetitive working or sport activities, but also hormonal and metabolic factors have been taken into account as predisposing conditions for the onset of tendon disorders [2]. The characteristic histopathological features of tendinopathy are the accumulation of fat cells, mucoid degeneration, tissue calcification, or combinations of all [1, 2]. Therefore, tendons contain cells with the potential to exhibit multiple phenotypes that differ from tenocytes, which express the fibroblast phenotype [2, 4]. This suggests that there is a complex etiopathogenetic mechanism underlying tendon damage. These findings also reflect a failure in the native tendon repair process: regarding this issue, the progress made in the field of tissue engineering and regenerative medicine (TERM) may represent a promising approach for the treatment of these challenging lesions [5, 6].

Among treatment strategies, injective treatments are commonly adopted, with “traditional” agents such as hyaluronic acid [7] or new biological products, such as platelet-rich plasma [8–10], autologous fibroblasts, tenocytes, and even mesenchymal stem cells [11] (MSCs). There is a growing evidence advocating the use of MSCs: the key feature of such cells is their capability to proliferate and differentiate into several cell lines, hence promoting tissue healing and repair [12]. MCSc can be obtained from different sources, but in routine clinical practice, bone marrow and adipose tissue are the most common choices [13]. Their manipulation involves expansion in the laboratory, enzymatic digestion, or even “minimal handling” consisting in centrifugation directly in the operating theatre. The products obtained by these processes can be injected locally or even adopted as an augmentation to biomaterials [13, 14]. The biological pinnacle of this treatment option is related to the particular mechanisms regulating tendon healing: a process consisting of different phases (inflammation, formation, and remodeling) characterized by the sequential expression of different growth factors (GFs) and other molecules playing a crucial role in tissue maturation: cells are able to upmodulate the overall tissue healing response, by stimulating anabolic processes and restoring tissue homeostasis [15].

The aim of the present scoping review is to summarize the available clinical evidence on the application of cell-based therapies to treat the most common upper limb tendinopathies, i.e., elbow and rotator cuff tendinopathies. Our review has therefore the following goals: (1) highlighting the current application methods, (2) describing the clinical outcomes of this biologic approach, and (3) understanding current limitations and areas of uncertainty that need to be elucidated by future researches.

## 2. Materials and Methods

A review of the literature was performed on the use of cell-based therapies to treat tendon disorders of the shoulder and elbow. The search was made on the PubMed and Scopus databases on December 2020, using the following formulas:

1. To identify clinical studies regarding rotator cuff tendinopathy: (rotator cuff tear OR partial rotator cuff tear OR rotator cuff repair) AND (cells OR stem OR MSCs OR bone marrow aspirate or BMAC or BMC OR fibroblasts OR tenocytes)
2. To identify clinical studies regarding elbow tendinopathies: (Epicondylitis OR Epicondylosis OR tennis elbow OR Elbow Tendinosis) AND (stem cells OR MSCs OR bone marrow aspirate or BMAC or BMC OR fibroblasts OR tenocytes)

**Figure 1:**
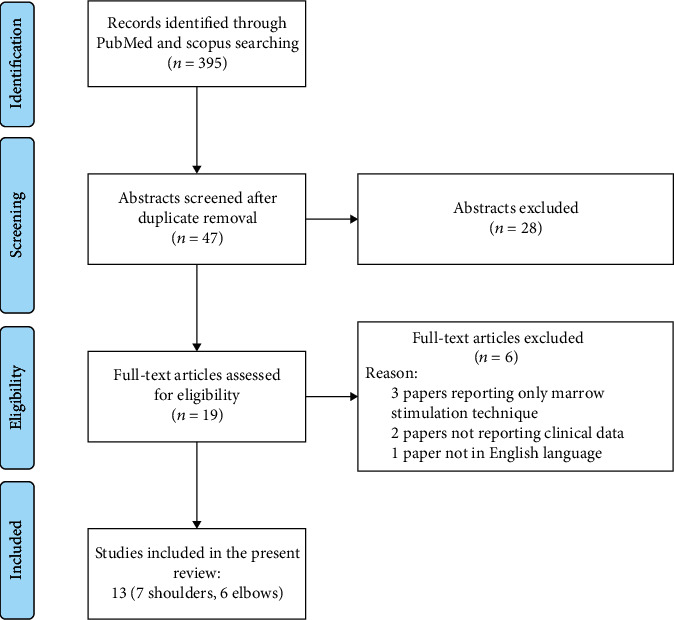
Flowchart resuming the paper's selection process for the present review.

The screening process and analysis were performed separately by 2 independent researchers (RR and AM). First, the articles were screened by title and abstract. The following inclusion criteria for relevant articles were used during the initial screening of titles and abstracts: clinical reports of any level of evidence, written in the English language, with no time limitation, on the use of MSCs or other cell-based approaches to treat rotator cuff or elbow tendinopathies, including cases of biological augmentation during surgical procedure. Exclusion criteria were instead as follows: case reports or mini case series (<5 patients), articles written in other languages, and reviews. In the second step, the full texts of the selected articles were screened, with further exclusions according to the previously described criteria. Moreover, articles not reporting clinical results were excluded. Reference lists from the selected papers were also screened. A PRISMA [16] flowchart of the selection and screening method is provided in Figure 1.

Relevant data were then extracted and collected in a single database with the consensus of the two observers to be analyzed for the purposes of the present manuscript. In particular, the following data were retrieved: (1) study design, (2) sample size, (3) delivery method, (4) eventual concurrent treatments (surgery or other substances), (5) outcome measures and timepoints of follow-up evaluations, and (6) summary of clinical results. Any discrepancy was discussed with and resolved by the senior investigator (AC), who made the final judgement. The primary outcome of the present scoping review was the variation in patients' reported subjective scores and pain evaluation in order to understand if the cell-based approach may provide any clinical benefit.

Furthermore, a quality assessment of each included trial was done by using the modified Coleman Methodology Score [17].

## 3. Results

Thirteen studies [18–30] were included in the present analysis: seven studies focused specifically on rotator cuff tendinopathies [18–24], whereas six studies [25–30] on elbow tendinopathies.

### 3.1. Methodology Assessment

The assessment through the modified Coleman score (range: 0–100) revealed modest results for all the trials analyzed, mainly due to the low number of patients included, the short follow-up, and the frequent presence of concurrent treatments, i.e., surgery or other substances used, thus resulting in a bias to the understanding of the contribution of cell-based approaches. The average scores were as follows: 54.4 (range 47–64) for rotator cuff studies and 47.7 (range 47–52) for elbow tendinopathy studies. The individual score for each included trial has been reported in Tables 1 and 2.

### 3.2. Qualitative Synthesis of Clinical Results

#### 3.2.1. Rotator Cuff Pathology

Seven papers in total met the inclusion criteria and were analyzed [24–30]. The most relevant features of each study have been summarized in Table 1. Three trials analyzed the effect of MSCs (bone marrow derived in 2 studies and adipose derived in one) as augmentation during rotator cuff surgical repair, whereas four trials (3 bone marrow derived and 1 adipose derived) investigated the effect of simple injections of MSCs for rotator cuff tears **(**Table 1). Only two trials reported data at follow-up longer than 12 months.

Looking at MSCs in association with surgical repair, two comparative studies [18, 19] found improvement in terms of healing and retear rates with the use of MSCs compared to the control group. In particular, Hernigou et al. [18] analyzed the healing within six months and the retear rate at ten years with MRI and ultrasound imaging in patients treated by single-row repair of full-thickness supraspinatus tear (<3 cm in the anteroposterior dimension), augmented or not with bone marrow aspirate concentrates (BMAC) harvested from the iliac crest. At six months, they found 100% of healing with MSC augmentation versus 67% of the control group; at ten years, intact rotator cuffs were found in 87% of MSC-treated patients, but just in 44% of the control patients. Moreover, authors found that the number of transplanted MSCs correlated with the healing rate: those who failed received overall a significantly lower amount of MSCs (14000 ± 9000 vs 54000 ± 23000, respectively), thus revealing a remarkable variability in the biologic properties of the bone marrow harvested. Kim et al. [19], in their matched case control study, analyzed the effect of adipose-derived MSCs in association to double-row surgical repair for medium to massive full-thickness rotator cuff tear, and they found a lower retear rate with the use of MSCs at 14 months, even if at the last follow-up, there was no significant difference between the two groups in terms of Constant score, UCLA score, pain, and range of motion (ROM). Another case series [20] reported good functional results (UCLA score improvement from 12 to 31) and 100% tendon integrity at one year, combining mini-open rotator cuff repair to biologic augmentation with BMAC from the iliac crest.

Regarding the use of simple MSC injections, there are only one case-control study and three case series. Kim et al. [21] compared the efficacy of BMAC + PRP injection for partial rotator cuff tear to a control group of patients assigned to physical therapy. At 3 months, pain and ASES scores were significantly better in the injection group and BMAC-PRP application contributed to decrease the size of the tear, although no significant difference compared to the control group was detected in this parameter. Regarding the other trials, the same group of Kim et al. [22] reported a previous case series of patients treated by BMAC + PRP injection for partial rotator cuff tear, with similar encouraging results; furthermore, the same paper presented also *in vitro* results that provided the rationale for combining BMAC and PRP: their combination, in fact, enhanced proliferation and migration of tendon-derived stem cells, preventing their aberrant chondrogenic and osteogenic differentiation. Centeno et al. [23] evaluated the effects of BMAC + PRP + platelet lysate (PL) injection for rotator cuff tear (partial or full thickness), in patients followed with an electronic database system. Despite the high rate of patients lost to follow-up, DASH and pain improved significantly at 7–8 months' follow-up. Jo et al. [24] in a prospective 2-step study evaluated the safety and efficacy of intratendinous injection of autologous adipose tissue-derived MSCs (a-dMSCs) in 18 patients with symptomatic partial rotator cuff tear. They used 3 different doses in 3 groups of three patients each in the first step of the study in order to assess the safety of the procedure, and then, 9 patients were administered the highest dose. They found that the injection of a-dMSCs led to improvement in SPADI, Constant score, and pain and also in MRI tendon defect at 6 months and that the improvement was related to the amount of MSCs: the high-dose group showed the best results in terms of clinical outcome, pain, and imaging. The authors also performed arthroscopic second look evaluations at 6 months, which revealed tendon regeneration and a significant decrease in tear volume for bursal-sided lesions (high-dose group) and for articular-sided defects (mid-dose group). No major adverse events were described.

#### 3.2.2. Elbow Tendinopathies

Six papers in total met the inclusion criteria and were analyzed [25–30]. The most relevant features of each study have been summarized in Table 2. All the trials were case series and five presented follow-up equal or less than 12 months. Three of them dealt with bone marrow-derived MSCs whereas the other three involved dermal fibroblasts and autologous tenocytes. The first use of stem cells in elbow tendinopathy was described by Moon et al. [25]. They used BMAC in combination with arthroscopic debridement in 26 elbows affected by lateral or medial tendinopathy, reporting a significant improvement in functional results and pain, with healthy tendon ultrasound appearance at 6 months follow-up. In another study, Singh et al. used BMAC containing also PRP in 30 patients with lateral epicondylitis and found improvement in Patient-Rated Tennis Elbow Evaluation (PRTEE) scores at 2, 6, and 12 weeks after treatment [26]. In a small study on 12 patients, Lee et al. treated chronic lateral epicondylitis with injections of allogeneic adipose-derived MSC with two different dosages of cells: they found a progressive improvement in pain, function, and ultrasound imaging up to the study final evaluation at 52 weeks, with no major adverse events reported, and slightly faster recovery in the high-dose group [27]. The other 3 studies did not employ mesenchymal stem cell therapy: Connell et al., in a pilot study on 12 patients, evaluated the effect of injections of collagen-producing cells derived from dermal fibroblasts: the authors documented no adverse effect, clinical improvement, and increased tendon thickness at 6 months [28]. Similar findings were reported by Wang et al. in two studies regarding the same cohort of 16 patients with refractory lateral epicondylitis: by using injections of autologous tenocytes derived from patellar tendon cells, they obtained a significant improvement in pain, functional score, and tendon appearance at MRI at 1 months, 3 months, 6 months, and 1 year [29], with stable results up to five years follow-up and 93% of overall patient satisfaction [30].

## 4. Discussion

The main finding of the present scoping review is the lack of high-level literature regarding the application of cell therapy in the management of tendinopathies involving both shoulder and elbow, thus making it very hard for clinicians and researchers to clearly understand the role of this biological approach to treat these degenerative injuries. Up to the present, 13 studies have been published and most of them are case series, with overall modest methodological quality, as revealed by the modified Coleman score, mainly due to the low number of patients included and the heterogeneity of procedures and therapeutic protocols adopted. Due to these limitations, it was not possible to perform a sounding quantitative analysis of the data extracted from the included trials.

Considering rotator cuff tendinopathy, even if all the reports suggest a favorable role of MSCs in stimulating tendon healing and symptomatic relief [18–24], it is essential to differentiate the application of stem cells as an isolated injective therapy or as augmentation during surgical repair. In particular, the preliminary evidence available suggests that MSCs could contribute to reduce the retear rate following cuff repair [18, 19]. Interestingly, Hernigou et al. also found that the number of transplanted MSCs correlated with the clinical outcome: in fact, concentration of MSCs exceeding 2500 cells per ml provided superior outcomes [18]. This suggests that the concentration of the stem cells might be a key factor for the results, but at the moment, there is lack of data on the ideal stem cell concentration to be applied, also considering that, even if the injection of MSCs is performed with closed pump, some liquid can remain in the shoulder, thus diluting the injected product, and cells may also migrate over the subacromial space. The injection of MSC combined with a biocompatible scaffold may help in solving this problem, as suggested by Kim et al. [19]. With regards to the use of MSC injections as isolated therapy, the level of evidence is very low and many limitations emerged [21–24]: in particular, the study of Centeno et al. [23] is affected by a very high percentage of patients lost to follow-up, and moreover, the concomitant use of MSCs + PRP + platelet lysate prevents from understanding the real contribution of MScs. Other 3 studies specifically evaluated the effect of MCS injections for partial rotator cuff tear. The group of Kim et al. in 2 studies [21, 22] combined MSC and PRP in the same injections reporting improved functional results, even if the ultrasound did not reveal a significant change in the tear size. In their 2017 paper [22], they performed also an in vitro evaluation which provided the rationale for combining MSC and PRP for tendinopathy: they demonstrated that this combination enhanced the proliferation and migration of tendon-derived stem cells, preventing at the same time their aberrant chondrogenic and osteogenic differentiation. In the last trial, authored by Jo et al. [24], it was found again that higher doses of MScs (10^8^ cells) provided the best outcomes: despite the small sample size of the study, this finding fosters further research in the dose-response field. Interestingly, this is also the only study where second-look arthroscopy was performed, showing glossy, white, and smooth appearance of the regenerated tendon fibers. Regarding cell therapy for elbow tendinopathies, even in this case, the overall level of evidence is very low with only case series available [25–30]. All the clinical studies reported a positive outcome in stimulating tendon healing and symptomatic relief at short- to medium-term follow-up, with no major complications related to the treatment. One study evaluated the effect of MSC in combination with arthroscopic debridement: even if the authors reported good results, the efficacy of adding MSC is questionable because there was no control group and arthroscopic debridement alone is considered an effective treatment for lateral epicondylitis [25]. Three studies used other cell therapy approaches: Connell et al. evaluated the effect of injection of collagen-producing cells obtained from dermal fibroblasts and prepared in the laboratory [28]. Wang et al. used expanded autologous tenocytes obtained from patellar tendon and injected in the elbow, reporting results at 1 year and then up to 5 years follow-up [29, 30]. Lastly, Lee et al. investigated the injection of allogeneic adipose-derived MSC harvested from human subcutaneous fat tissue of healthy donors [27]. They reported only a minor degree of elbow joint effusions in 2 subjects, without serious major side effects. They also used two different doses of stem cells, underlining that higher concentration of stem cells tended to induce earlier clinical improvement. As for the shoulder, there is not a clear definition of the optimal dose of stem cells for treating chronic elbow tendinopathy, even if this study leads another evidence that stem cell concentration has a relevant role for the outcome. This study, moreover, is the only one to use allogenic stem cells: that might represent an advantage in terms of availability and reduced morbidity for the patients, but ethical considerations and the lack of data on long-term safety prevent the routine use of allogeneic stem cells.

Despite the well-established rationale for the use of cell-based therapies, as testified by several preclinical in vitro and in vivo studies, current clinical literature offers very low and weak evidence and randomized controlled trials appear necessary in the near future.

Besides considerations regarding the level of evidence of the available studies, other controversial aspects should be acknowledged. First of all, the marked interproduct variability and the different application strategies must be underlined. In fact, similarly to other biologic approaches [31], cell-based therapies can widely vary according to the following: site of harvesting, production technique, cellularity, injections with scaffold or other additives, timing, and number of applications. All these variables make it very hard to identify the best formulation to adopt for the treatment of tendinopathies, and current clinical data did not reveal any correlation between specific product features and clinical outcome, with the only likely exception of cell count. Regarding the sites of harvesting, it was proved that the morphology and immune phenotype of the MSCs derived from bone marrow or adipose tissue are the same [32, 33]. However, in vitro studies showed that adipose-derived MSCs are viable for a longer time and exhibit higher proliferation rates and higher density [33–35]. So, theoretically, adipose-derived MSC may have some “biologic” advantages, but their superiority in the clinical setting has yet to be demonstrated [13]. Another relevant aspect is the role of concurrent treatments, in particular surgery, which always represent a traumatic stress for joints, even in case of arthroscopic procedures. In fact, on one side, MSCs are supposed to mitigate the inflammatory response following surgery and promote tissue healing, but on the other side, surgery itself might reduce the regenerative potential of MSCs due to the increase in the inflammatory distress induced within the articular environment [36]. Based on these premises, the real role of cells in the clinical setting should be better studied without the “interference” of surgery. Even the adoption of other bioactive substances (in particular PRP, often injected together with MSCs) may represent another confounding variable, despite preliminary demonstrations of a synergic action of these two biologic agents also in other orthopaedic diseases [37]. In this scenario, it appears clear that the identification of the “optimum” cell-based therapeutic strategy is far from being reached, but perhaps, this is not the “current” crucial point in this particular field of research, which is still affected by a huge burden of regulations and ethical limitations in many countries [38]: all the reports included in the present review, dealing not only with MSCs but also with other less common sources (fibroblasts and tenocytes), suggest a beneficial role of cell-based therapies in the management of tendinopathies that needs to be confirmed by randomized controlled trials including not only clinical but also proper imaging evaluations at middle- to long-term follow-up. The current lack of high-level evidence is partly due to regulatory and ethical restrictions, especially in the US and Europe, and partly to the high-cost inherent to the use of cell-based approaches [13]. In terms of expenses, the comparison among cell-based and “traditional” strategies is clearly unfavorable for the former but, when dealing with degenerative pathologies such tendinopathies, the real game changer approach is not aimed at providing temporary symptomatic relief but at reducing the relapse rate. In fact, there are many current strategies to reduce pain in elbow tendinopathies but none of them has shown long-term durability, and even looking at rotator cuff repair for full thickness tears, although satisfactory outcomes have been reported, the retear rate is still a relevant concern for surgeons and patients [3]. Improving the biology of tendon healing could represent the strategy to extend the duration of beneficial effects, minimize failures, and therefore reduce the need for retreatment and the inherent costs over time.

## 5. Conclusion

The use of cell-based approaches for treating elbow and rotator cuff tendinopathies showed overall safety and positive preliminary clinical findings. The most commonly adopted strategy entails the use of autologous MSCs harvested from bone marrow, but even fibroblasts and tenocytes have been tested with good outcomes. Cells can be injected locally or even applied as an augmentation during the surgical procedure, but despite encouraging clinical results, current data does not allow to endorse the routine use of cell-based approaches and well-designed RCTs are needed to confirm their real therapeutic efficacy against traditional options.

## Figures and Tables

**Table 1:** Synopsis of clinical studies dealing with cell-based approaches in rotator cuff pathology.

Publication	Level of evidence	Modified Coleman score	Pathology	*N* of patients	Therapeutic protocol	MSC manipulation	Outcomes and imaging	Follow-up	Results
WITH ROTATOR CUFF SURGICAL REPAIR									
Ellera Gomes et al., KSSTA [20]	IV case series	58	Full-thickness tear	14	Transosseous mini *open* + *BMAC* form post iliac crest (same time of surgery)	100 ml of bone marrow: MSC fractions were obtained according to good manufacturing practices by Ficoll–Hypaque density gradient and then resuspended in saline solution enriched with 10% autologous serum to a final volume of 10 ml	*UCLA* + *MRI*	12 months	(i) Good functional results (ii) Tendon integrity in all cases at 12 months MRI with some signal artifacts which did not affect the final functional result (iii) 1 failure
Hernigou et al., Int Orthop.[18]	III case control study	64	Full-thickness supraspinatus tear (1.5–2.5 cm)	90 (45 with and 45 without ASC)	Arthroscopic single-row repair with suture anchors with or without BMAC from anterior iliac crest (same time of surgery)	150 ml of bone marrow aspirate concentrated in the cellular and molecular therapy laboratory	US (every month), MRI (3 months, 6 months, 1 year, 2 years, and last minimum 10 years)	Minimum 10 years	(i) MSCs improved the healing rate at 6 months and decreases the retear rate at 10 years (ii) The number of MSCs correlated to the grade of healing
Kim et al., AJSM [19]	III cohort study	63	Full-thickness RCT	70 (35 with and 35 without ASC)	Arthroscopic double-row repair with or without a-dMSC (from the *buttock*) + *fibrin* glue scaffold (liposuction one day before arthroscopy)	Liposuction of 120 ml of adipose tissue: MSC lab. isolation and preparation followed by injection with fibrin glue scaffold—Greenplast kit (Green Cross)	VAS, CS, UCLA, and*ROM* + *MRI* (minimum 1 year)	Minimum 24 months	(i) No significant functional difference (ii) Significantly lower retear rate with MSCs
WITHOUT ROTATOR CUFF SURGICAL REPAIR									
Centeno et al., Journal of Pain Research [23]	IV case series	52	G-H OA and/or partial or full-thickness tear	^∗^Uncontrolled treatment registry population: 115 (81 RCT and 34 OA); available follow-up for 40 (DASH) and 55 (NPS)	BMAC from post iliac *crest* + *PRP* and PL injection (+hypertonic dextrose solution injection 2 or 5 days before) intra-articular or in the RCT	BMAC centrifugation followed by addition of PRP and PL	DASH, NPS	3 and 24 months	(i) Significant improvement of DASH and pain (NPS) (ii) No differences between OA and rotator cuff groups
Kim et al., Cell Transplantation [22]	IV case series	47	Partial thickness tear	12	BMAC from iliac *crest* + *PRP* injection at the tear site (US guidance)	BMAC centrifugation with BIOMET MarrowStim™ mini kit followed by injection of 2 ml of BMACs mixed with 1 ml of PRP	ASES, *VAS* + *US*	3 months	(i) Significant improvement of VAS and ASES (ii) Reduction of the tear size but the change was not statistically significant
Kim et al., JOSR [21]	III case control study	49	Partial thickness tear	24 (12 BMAC-PRP vs 12 rehabilitation)	BMAC from iliac *crest* + *PRP* injection at the tear site (US guidance)	BMAC centrifugation with BIOMET MarrowStim™ mini kit followed by injection of 2 ml of BMACs mixed with 1 ml of PRP	ASES, *VAS* + *US*	3 months	(i) Significantly higher VAS and ASES in the BMAC-PRP group (ii) The change in the tear size did not differ significantly between groups (iii) Manual muscle test and use of medications were not significantly different between the two groups
Jo et al., Stem Cells [24]	IV case series	48	Partial thickness tear	18 (3 low, 3 mid, and 3 high dose for safety review and then the other 9 pts are high dose)	Injection of a-dMSC (from abdomen) (liposuction 3 weeks before injections)	Cells from stromal vascular fraction isolated and cultured in keratinocyte-SFM- (Invitrogen) based media containing 0.2 mM ascorbic acid, 0.09 mM calcium, 5 ng/ml recombinant epidermal growth factor, and 5% fetal bovine serum	SPADI, CS, *VAS* + *MRI* (1 month, 3 months, and 6 *months*) + *arthroscopic* examination	6 months	(i) No serious adverse event (ii) SPADI and CS significantly improved in mid- and high-dose groups (iii) VAS significantly alleviated by 71% in the high-dose group (iv) Bursal side defect significantly decreased by 90% in the high-dose group at MRI examination (v) Articular and bursal side defects decreased by 83% and 90% in the mid- and high-dose groups at arthroscopic examination

RCT: rotator cuff tear; G-H OA: gleno-humeral osteoarthritis; BMAC: bone marrow aspirate concentrate; MSC: mesenchymal stem cells; a-dMSC: adipose-derived mesenchymal stem cells; PRP: platelet-rich plasma; PL: platelet lysate; VAS: visual analogue scale; NPS: numeric pain scale; CS: Constant-Murley Score; ASES: American Shoulder and Elbow Surgeons; UCLA: University of California, Los Angeles; DASH: shoulder and hand score; SPADI: Pain And Disability Index; ROM: range of motion; MRI: magnetic resonance imaging; US: ultrasound.

**Table 2:** Synopsis of clinical studies dealing with cell-based approaches in elbow tendon pathology.

Publication	Level of evidence	Modified Coleman score	Pathology	*N* of patients	Therapeutic protocol	MSC manipulation	Outcomes and imaging	Follow-up	Results
Moon et al., Ann Acad Med Singapore [25]	IV case series	48	Medial and/or lateral epicondylitis	24 (26 elbows)	Arthroscopic *debridement* + *BMAC* from anterior Iliac crest	20 ml of bone marrow plasma was centrifuged and kept in the refrigerator. Only the clear upper layer and the buffy coat layer were used obtaining 8 to 9 ml mixed with 3 ml of bupivacaine before injection	VAS, *MEPS* + *US*	8 weeks and 6 months	(i) Statistically significant improvement in VAS and MEPS (ii) No complications, 2 failures (iii) Healthy tendon appearance at US at 6 months
Connell et al., Br J Sports Med [28]	IV case series (pilot study)	45	Lateral epicondylitis	12	Injection of collagen-producing cells from dermal fibroblasts	4 mm skin sample obtained from the lateral side of the hip. Lab preparation of the cells and injections of 10^6^ *x* 10^6^ collagen-producing cells (approximately 2 ml)	*PRTEE* + *US*	6 weeks, 3 weeks, and 6 months	(i) Statistically significant improvement in PRTEE (ii) No complications, 1 failure (iii) Healthier tendon appearance at US in terms of tendon thickness, hypoechogenicity, intrasubstance tears, and neovascularity at 6 months
Wang et al., AJSM [30]	IV case series	47	Lateral epicondylitis	18	Injections of expanded autologous tenocytes from patellar tendon	A 3 *x* 1 *mm* strip of tendon was harvested from the superficial surface of the patellar tendon. Tenocytes were cultivated and up to 2 ml of autologous tenocytes (2–5 *x* 10*^*6 cells/ml) suspended with 10% autologous human serum were injected after 3 weeks	VAS, qDASH, grip strength, +MRI score (12 months)	1, 2, 3, 12, 12 months	(i) Statistically significant improvement in VAS score, qDASH, grip strength score, and MRI scores after surgery and at last follow-up (ii) No major complications at donor or injection site (iii) 2 patients withdrew and had surgery
Singh et al., J Nat Sci Biol Med [26]	IV case series	45	Lateral epicondylitis	30	BMAC from iliac crest	10 ml of bone marrow plasma was centrifuged; only the clear upper layer and the buffy coat layer was used obtaining 4 to 5 ml mixed with 1 ml of 2% lignocaine solution before injection	PRTEE	2 weeks, 6 weeks, and 3 months	(i) Statistically significant improvement in PRTTE (ii) No major complications
Lee et al., Stem Cells [27]	IV case series	49	Lateral epicondylitis	12	allo-adMSC mixed with fibrin glue injection	Lipoaspirates of human subcutaneous fat tissue obtained from healthy donors were treated in the lab obtaining allo-a-dMSC. All the procedures followed the “Cell Bank process”. Injections of 0.5 ml thrombin mixed with 10^6^ or 10^7^ (2 doses) of allo-ASCs in the first syringe and 0.5 ml fibrinogen in the other syringe	MEPI, *VAS* + *US*	2 weeks, 6 weeks, 12 weeks, and 52 weeks	(i) VAS and MEPI score significantly improved and tendon defect decreased at US over the course of the follow-up (ii) No significant adverse effects (iii) No significant differences between the two dose groups, even if faster pain improvement and earlier plateauing of performance scores were observed in the higher dose group
Wang et al., AJSM [30]	IV case series	52	Lateral epicondylitis	15	Injections of expanded autologous tenocytes from patellar tendon	As above	VAS, qDASH, UEFS, grip *strength* + *MRI* score	4 years, 5 years	(i) Significant improvements from preop were maintained in all clinical and MRI scores for up to 5 years after treatment (ii) At final follow-up, 93% of patients were either highly satisfied or satisfied; 1 patient (with tear progression) was not satisfied

BMAC: bone marrow aspirate concentrate; allo-adMSC: allogeneic adipose-derived mesenchymal stem cells; MEPS: Mayo Elbow Performance Scoring; PRTEE: Patient-Rated Tennis Elbow Evaluation; qDASH: Quick Disability of the Arm, Shoulder, Hand Score; VAS: visual analog scale; UEFS: upper extremity functional scale; MRI: magnetic resonance imaging; US: ultrasound.

## Data Availability

All the data analyzed for the purpose of the review have been already included into the manuscript.
